# Comparison of Energy and Frequency of Nanosecond Q-switched ND-YAG Laser and Chlorhexidine on Reducing the Number of Oral Streptococcus Mutans Isolates: In Vitro Study -

**DOI:** 10.31661/gmj.vi.3973

**Published:** 2025-11-18

**Authors:** Faramarz Zakavi, Nazanin Kazemi, Azita Kaviani, Mohammad Sabaeian, Mohammad Hashemzadeh

**Affiliations:** ^1^ Department of Restorative Dentistry, School of Dentistry, Ahvaz Jundishapur University of Medical Sciences, Ahvaz , Iran; ^2^ Center for Research on Laser and Plasma, Shahid Chamran University of Ahvaz, Iran; ^3^ Department of Microbiology, School of Medicine, Ahvaz Jundishapur University of Medical Sciences, Ahvaz, Iran

**Keywords:** Streptococcus Mutans, Chlorhexidine, Nd:YAG Nanosecond

## Abstract

**Background:**

Streptococcus mutans is a facultative anaerobic coccus that is a part of the oral flora of humans. chlorhexidine also has some side effects. Today, Nd: YAG lasers have become very popular in dentistry and are used for various types of treatment. Therefore, this study aims to compare the effects of Chlorhexidine 2% and nanosecond (Nd: YAG) laser in reducing the number of oral Streptococcus mutans bacteria in the oral cavity.

**Materials and Methods:**

An in vitro experimental design was conducted using S. mutans ATCC 35668 cultured on Mueller-Hinton agar. Bacterial suspensions were standardized to a half-McFarland turbidity and distributed in 96-well plates. The antimicrobial activity of 2% CHX was assessed via the well-diffusion method. Laser irradiation was applied at varying energies (10, 20, 30 mJ) and frequencies (5, 10 Hz). Bacterial counts and inhibition zone diameters were measured, and data were analyzed using two-way ANOVA followed by Tukey post hoc tests (α=0.05).

**Results:**

Bacterial counts decreased with increasing laser energy and frequency, with complete elimination observed at 30 mJ (both frequencies) and at 20 mJ/10 Hz. Inhibition zone diameters were largest at 30 mJ/10 Hz (8.6 ± 0.39 mm), while 2% CHX produced a slightly larger, but not statistically significant, inhibition zone (13 ± 2.9 mm vs. 11.5 ± 14.4 mm; P.05). Both laser parameters and CHX significantly reduced S. mutans populations, demonstrating comparable antimicrobial efficacy at higher laser energies.

**Conclusion:**

High-energy 1064 nm Q-switched Nd:YAG laser therapy effectively reduces S. mutans counts in vitro, with results comparable to 2% CHX. Laser application may serve as an alternative or adjunctive strategy for microbial control in dental treatments, particularly in cases where chemical antiseptics are limited or contraindicated.

## Introduction

The human oral cavity provides a highly favorable environment for the continuous formation of natural microbial biofilms. Nevertheless, when the balance of oral health is disrupted, pathogenic microorganisms can invade dental tissues and the gingival region. These infectious pathogens originating from the oral cavity are responsible for oral diseases such as caries, periodontitis, root infections, and alveolar osteitis, and they may also be linked to systemic conditions including cardiovascular disorders, stroke, preterm birth, diabetes, and pneumonia [2][3].

Among these microorganisms, Streptococcus mutans is one of the most significant oral bacteria, playing a key role in plaque formation and acting as a major contributor to dental caries, which can consequently impact overall host health. This bacterium creates an acidic environment in the mouth by metabolizing various carbohydrates, and elevated levels of S. mutans place an individual at a high risk for caries [4].

Chlorhexidine gluconate (CHX) is commonly employed as a broad-spectrum antibacterial antiseptic, effective against Gram-positive and Gram-negative bacteria, fungi, and some viruses, and it is known to inhibit the formation and progression of bacterial plaque for several hours [5][6]. Studies indicate that CHX concentrations ranging from 0.12% to 2% can effectively suppress matrix metalloproteinase (MMP) activity, thereby enhancing the long-term bond strength between restorative surfaces and dentin. Moreover, using CHX at concentrations between 0.12% and 0.2% significantly reduces bacterial indices, inflammation, and gingival bleeding [6][7].

It is generally accepted that to achieve maximal microbial reduction and maintain an oral cavity as free of bacteria as possible, a combination of mechanical preparation and irrigation with copious amounts of detergent is most effective. Ideally, a detergent should not only assist in the mechanical cleaning process but also possess bactericidal and lytic properties. The main constituents of CHX are the acetate and hydrochloride salts, which exhibit limited solubility in water; therefore, chlorhexidine digluconate has been used as a more soluble alternative. As a potent disinfectant, CHX is widely applied for chemical control of the oral cavity, typically in aqueous solutions of 0.1% to 0.2%. In endodontics, 2% CHX has been conventionally used as a canal rinse [8][9] .

The advancement of laser medicine has introduced new therapeutic modalities capable of damaging pathogenic organisms, particularly where direct contact between the chemical agent and microorganism is essential for bactericidal effects [11]. Since the 1990s, lasers have gained popularity in dentistry for a variety of treatments [12]. Multiple studies have examined the bactericidal and antimicrobial potential of lasers, among which low-level laser therapy (LLLT) shows promise, as it delivers no thermal radiation to the specimen surface. Low-energy lasers (685 and 830 nm) are considered safe and effective in targeting biofilm-associated infections [13][14].

The neodymium-doped yttrium aluminum garnet (Nd:YAG) high-energy 1064 nm Q-switched laser, with output obtained through LD array end-pumping, exhibits high scattering and tissue penetration capabilities, making it effective in reducing levels of S. mutans and Candida albicans [15][16]. This laser exerts a photothermal effect, destroying bacteria through vaporization, structural destruction, or denaturation, leading to their inactivation [17]. The antibacterial efficacy of the Nd:YAG laser in patients with oral diseases has been confirmed in multiple studies. Additionally, desensitization strategies are frequently employed before restorative procedures in hypersensitive dentin, with Nd:YAG laser therapy being one such approach [18].

Therefore, the present study aims to compare the effectiveness of 2% Chlorhexidine and nanosecond high-energy 1064 nm Q-switched Nd:YAG laser output obtained by LD array end-pumping in reducing the population of S. mutans in the oral cavity.

## Materials and Methods

### Study Design and Setting

This in vitro experimental study was conducted in the Jundishapur Microbiology Laboratory, Ahvaz, Iran, to evaluate the effects of Nd:YAG laser and 2% chlorhexidine on Streptococcus mutans (ATCC 35668).

### Microbial Culture Conditions

After resuscitation of the lyophilized S. mutans strain ATCC 35668 (stored at −70°C) for preservation and stock maintenance, all procedures were conducted under aseptic conditions in the laboratory. The workbench was sterilized in two stages using sterile cotton and 70% ethanol. Microbial cultures were performed linearly on Mueller-Hinton agar to minimize environmental contamination, in proximity to a Bunsen flame and away from cooling devices.The culture procedure was as follows: initially, a portion of the culture was examined for contamination. If contamination was observed, the pure strain was isolated. During inoculation, the swab was pressed against the inner wall of screw-capped tubes to remove excess liquid, then streaked onto the agar surface in a zigzag pattern covering approximately 30% of the plate. The swab was then rotated 90° and streaked again across the initial section, continuing the zigzag pattern for 2-3 passes. This procedure was repeated, progressively spreading smaller amounts to generate isolated colonies. In the linear streaking method, the second section shows fewer colonies, while the final section produces predominantly isolated colonies. Plates were incubated inverted at 37°C for 24 hours and visually evaluated the following day.

### Preparation of Standard Half-mcFarland Turbidity for Bacterial Inoculation

A half-McFarland standard suspension (1.5 × 10^8 CFU/mL) was prepared by adding 5.0 mL of 0.045 M barium chloride (1.175% w/v BaCl2·2H2O) to 5.99 mL of 0.36 M sulfuric acid (0.18 M, 1% v/v) under continuous stirring. The turbidity was verified using a spectrophotometer at 625 nm with a 1 cm path length, targeting an absorbance range of 0.08-0.13. The barium sulfate suspension was aliquoted into 4-6 mL screw-capped tubes, stored in the dark at room temperature, and vortexed prior to each use. Any formation of large particles necessitated preparation of a new standard.

### Preparation of Bacterial Inoculum in 96-well Plates

Half-McFarland equivalent bacterial suspensions were distributed at 100 µL per well into 96-well plates, grouped into seven sets of 10 wells each. A separate 96-well plate was prepared for the 2% chlorhexidine control group (10 wells).

### Investigation of 2% Chlorhexidine Effects Using the Well-diffusion Method

The antimicrobial effect of 2% chlorhexidine on S. mutans was assessed using the well-diffusion method on Mueller-Hinton agar. Wells were created in the agar, and chlorhexidine was added. Zones of inhibition were measured after incubation, indicating bacterial susceptibility to the antimicrobial agent.

### Study of Nd:YAG Laser Effects on S. mutans

Bacterial samples were irradiated using a high-energy 1064 nm Q-switched Nd:YAG laser (Sairan Company, Iran Electro-Optic Industries, model GL5010-01). This solid-state pulsed laser emits pulses of 10, 20, or 30 mJ at frequencies of 5 or 10 Hz. One pulse per frequency corresponds to one pulse per second.

Laser application was performed by directing pulses at the bacterial plates through a 1064 nm total reflection mirror. Glass slides were used to reduce energy: two slides reduced energy from 30 to 20 mJ, and four slides reduced energy from 30 to 10 mJ. The laser beam entered the optical fiber at a 45° angle to the optical center, with a focus diameter of 3 mm. Protective eyewear was used due to the invisible infrared wavelength.

### Study Groups

The experimental groups were organized as follows: Group 1 received 2% chlorhexidine, Group 2 was treated with an Nd:YAG laser at an energy of 10 mJ and a frequency of 5 Hz, Group 3 received an Nd:YAG laser at 10 mJ and 10 Hz, Group 4 was exposed to an Nd:YAG laser at 20 mJ and 5 Hz, Group 5 received an Nd:YAG laser at 20 mJ and 10 Hz, Group 6 was treated with an Nd:YAG laser at 30 mJ and 10 Hz, and Group 7 received an Nd:YAG laser at 30 mJ and 5 Hz.

### Statistical Analysis

In this study, data are examined in two parts: descriptive and inferential. In the descriptive part, data were reported as mean ± standard deviation. In the inferential part, analysis of variance (ANOVA) was used to compare the mean values of the measured values. In the analysis of variance, the total data are separated into more than two parts based on the group factor (chlorhexidine or laser), and the mean data in different groups are compared with each other. If the changes between groups were greater than the changes within groups, it would indicate a significant difference in the reduction of the number of bacteria between the groups. In this study, the hypothesis of the analysis of variance research was taken as no difference between the means of the measurements. If the P<0.05, the null hypothesis was rejected, and a significant difference in the number of bacteria between the different groups was concluded. If the results of the analysis of variance showed that the average values examined for the different groups were significantly different from each other, then for a more in-depth study, the groups were compared in pairs. For this purpose, post hoc tests were used. All studies were performed with SPSS software at an error level of 0.05.

## Results

**Table:** Table1. Average S. mutans Counts and Inhibition Zone Diameters at Different Laser Energies and Frequencies (mean ± SD)

**Energy (mJ)**	**Frequency (Hz)**	**Bacterial Count (mean ± SD)**	**Inhibition Zone Diameter (mm, mean ± SD) **
10	5	14.5 ± 1.7	0 ± 0
	10	9.0 ± 1.3	0 ± 0
20	5	2.0 ± 1.6	0 ± 0
	10	0 ± 0	2.2 ± 1.12
30	5	0 ± 0	6.5 ± 0.17
	10	0 ± 0	8.6 ± 0.39

The effects of laser energy and frequency on S. mutans bacterial counts and the diameter of the inhibition zone were examined. Table-1 presents the mean ± standard deviation of bacterial counts and inhibition zone diameters at different laser energies (10, 20, 30 mJ) and frequencies (5 and 10 Hz).

**Figure-1:**
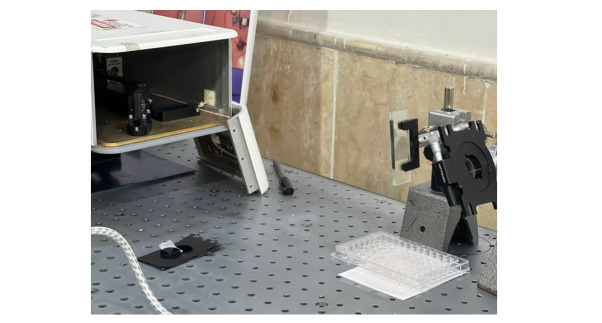
Figure 1. Reflecting mirrors

Bacterial Counts. As shown in Table-1, the number of S. mutans bacteria decreased with increasing laser energy at both frequencies. At 30 mJ, no bacteria were observed at either frequency. At 20 mJ and 10 Hz, bacterial growth was also absent. The highest bacterial counts were observed at 10 mJ and 5 Hz (14.5 ± 1.7). Two-way ANOVA revealed significant main effects of laser energy and frequency on bacterial counts (P<.001). Post hoc Tukey tests indicated significant differences between energy levels: 30 mJ (0 ± 0)<20 mJ (1.1 ± 1.2)<10 mJ (11.8 ± 2.9), and between frequencies: 10 Hz (3.4 ± 1.3)<5 Hz (5.6 ± 5.6) (P<.001 for all comparisons), shown in Figure-1.

**Figure-2:**
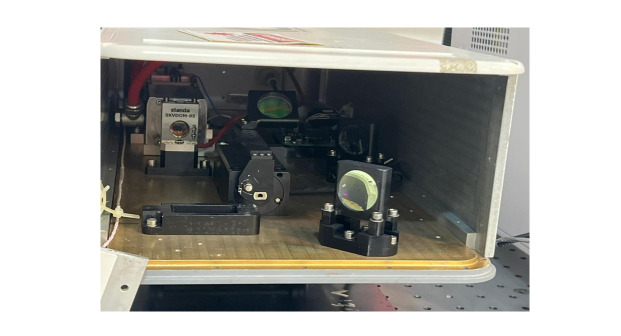
Figure 2. Laser output

**Figure-3:**
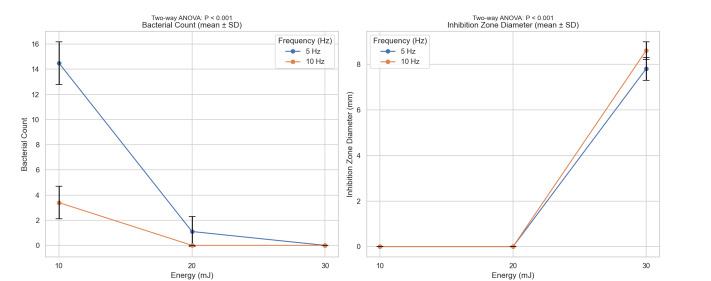
Figure 3. Bacterial counts stratified by Enerjy and frequency (left); inhibition zone sizes stratified by enerjy and frequency (right)

Diameter of the Inhibition Zone. Laser energy and frequency also significantly affected the diameter of the inhibition zone (P<.001). At 10 and 20 mJ, inhibition zones were minimal or absent at both frequencies. The largest inhibition zone was observed at 30 mJ and 10 Hz (8.6 ± 0.39 mm). Comparison with 2% chlorhexidine showed a slightly larger mean inhibition zone (13 ± 2.9 mm vs. 11.5 ± 14.4 mm for laser), but the difference was not statistically significant (P>.05), as shown in Figure-1.

## Discussion

This study evaluated the antimicrobial efficacy of a high-energy 1064 nm Q-switched Nd:YAG laser compared to 2% chlorhexidine (CHX) against Streptococcus mutans in an in vitro setting. The results demonstrated that both interventions significantly reduced S. mutans counts, with the laser achieving complete bacterial elimination at higher energy levels (30 mJ at 5 and 10 Hz, and 20 mJ at 10 Hz) and producing inhibition zones comparable to CHX (8.6 ± 0.39 mm at 30 mJ/10 Hz vs. 13 ± 2.9 mm for CHX, P>.05). These findings align with several studies that have explored Nd:YAG laser applications in reducing oral microbial loads, though differences in laser parameters, microbial targets, and experimental designs introduce variations that warrant deeper analysis.

The study by Grzech-Leśniak et al. (2024) [19] similarly investigated the Nd:YAG laser’s effect on S. mutans, Candida albicans, and Candida glabrata in both planktonic and biofilm forms, using lower irradiance settings (0.5 W/cm² for T1 and 1.75 W/cm² for T2) compared to our study’s higher energy densities (up to 0.597 J/cm² per pulse) [19]. Their results showed significant reductions in S. mutans (up to 85.4% in planktonic cultures and 94.3% in biofilms at T2 settings), consistent with our observation of complete bacterial elimination at higher energies. However, their effectiveness diminished after 24 hours, suggesting a temporary antimicrobial effect, whereas our study measured immediate post-irradiation effects, potentially explaining the sustained efficacy observed. The difference in laser parameters (lower power vs. our high-energy pulses) and the inclusion of biofilms may account for these variations, as biofilms are generally more resistant to antimicrobial treatments due to their complex extracellular matrix [20]. Our study’s focus on planktonic S. mutans likely facilitated greater bacterial susceptibility to laser energy, highlighting a key distinction in experimental design.

In contrast, Deeb et al. (2023) found that Nd:YAG laser alone had no significant effect on S. mutans, S. sanguinis, or E. faecalis, but its combination with 0.5% NaOCl or 0.12% CHX significantly enhanced bacterial reduction, particularly for S. mutans [21]. This diverges from our findings, where the laser alone was highly effective at higher energies. The discrepancy may stem from differences in laser parameters (unspecified energy densities in Deeb et al. vs. our 10-30 mJ pulses) and the lower CHX concentration used (0.12% vs. our 2%), which may have reduced standalone chemical efficacy in their study. Additionally, their inclusion of root caries-associated bacteria and broth-based cultures versus our agar-based well-diffusion method could influence bacterial response to laser treatment. The synergistic effect of laser with NaOCl in Deeb et al. [21] suggests potential for combined therapies, an area our study did not explore but could be considered for future research to enhance antimicrobial outcomes.

Anwer’s study [22] and another by Namour et al. (2021)[23] further corroborate the antimicrobial potential of Q-switched Nd:YAG lasers. Anwer reported significant S. mutans reductions at 0.796-0.955 J/cm² with 900-1260 pulses, aligning closely with our energy densities and pulse duration, reinforcing the dose-dependent bactericidal effect (Anwer, n.d.). Namour et al. (2021) achieved total disinfection of multi-species biofilms on titanium surfaces using similar parameters (0.597 J/cm², 10 Hz, 6 ns pulses), mirroring our complete elimination at 30 mJ [23]. However, their focus on titanium surfaces and multi-species biofilms contrasts with our single-species agar-based setup, suggesting that laser efficacy may be consistent across different substrates but varies with microbial complexity. The principle of selective photothermolysis, noted in the third referenced study, likely explains the laser’s targeted antimicrobial action in both our study and Namour et al. [23], where energy is preferentially absorbed by bacterial cells, minimizing damage to surrounding materials [24].

Despite these consistencies, limitations in our study and the referenced works highlight areas for further investigation. Our in vitro design, while controlled, does not replicate the complex oral environment, including saliva, temperature variations, and biofilm dynamics, which may reduce laser efficacy in vivo, as suggested by Grzech-Leśniak et al.’s reduced effect after 24 hours. The lack of biofilm testing in our study limits direct comparison with studies like Namour et al. [20] and Grzech-Leśniak et al. [19], where biofilms posed greater resistance. Additionally, our study did not assess long-term effects or combine laser with other antimicrobials, unlike Deeb et al. [21], potentially missing synergistic opportunities. Future research should explore in vivo applications, longer-term microbial regrowth, and combined laser-chemical protocols to validate clinical relevance. Synthesizing these findings, the Nd:YAG laser shows promise as an effective antimicrobial tool, particularly at higher energies, with potential as an alternative or adjunct to CHX, though its efficacy may depend on microbial type, culture conditions, and laser parameters.

## Conclusion

The results showed the superiority of laser over chlorhexidine in eliminating S. mutans bacteria, and it is necessary to conduct in vivo studies to investigate the safety of laser so that it can be used in the treatment of patients suffering from caries and other oral and dental diseases, as well as to prevent the formation of biofilm plaque. The advantages of the present study include the novelty of the subject, the accuracy in standardizing the laboratory study conditions (temperature, constant concentration of microbial suspension, uniform microbial distribution technique, etc.), and a sufficient number of samples to record and report the actual results.

## Conflict of Interest

The authors declare that they have no competing interests.
